# Segmented filamentous bacteria-based treatment to elicit protection against *Enterobacteriaceae* in Layer chickens

**DOI:** 10.3389/fmicb.2023.1231837

**Published:** 2023-07-31

**Authors:** Jared Meinen-Jochum, Logan C. Ott, Melha Mellata

**Affiliations:** ^1^Interdepartmental Microbiology Graduate Program, Iowa State University, Ames, IA, United States; ^2^Department of Food Science and Human Nutrition, Iowa State University, Ames, IA, United States

**Keywords:** segmented filamentous bacteria, *Enterobacteriaceae*, antimicrobial peptides, cytokines, layer chickens

## Abstract

**Introduction:**

Gut microbes like segmented filamentous bacteria (SFB) play a key role in gut maturation during early life, as demonstrated in humans and mice. Our previous study demonstrated oral inoculation of ileum-spores containing SFB to chickens after hatch increases early SFB gut colonization, which increases immune maturation and resistance to bacteria, like *Salmonella*, as tested *in vitro*; however, more studies are needed for treatment optimization and *in vivo* testing. The objectives of this study were to (1) test a treatment that includes both spores and filamentous SFB, (2) validate antimicrobial ability of the treatment in layer hens *in vivo,* and (3) elucidate its molecular mechanism.

**Methods:**

One-day-old specific pathogen-free layers (*n* = 12 per group) were orally treated with either PBS (CON) or SFB-based treatment (SFB). At 4 days post-inoculation (DPI), both CON and SFB groups were orally challenged with *Salmonella* Typhimurium. Total *Enterobacteriaceae* and *Salmonella* were examined by plating and enumeration in feces at 7,10 and 14 dpi; and in the ileum, cecum, and spleen at 16 dpi in euthanized birds. The presence and levels of SFB were determined from ilea scrapings *via* microscopy and qPCR, respectively. Relative gene expression of host-derived antimicrobial peptides and cytokines in the distal ileum was determined by RT-qPCR.

**Results:**

At 10 and 14 dpi, a significant decrease in total *Enterobacteriaceae* was observed in the feces of the SFB group. At necropsy, the level of SFB was significantly higher in the SFB group than in the CON group, while a significant decrease in total *Enterobacteriaceae* and *Salmonella* was observed in the ceca of the SFB group. RT-qPCR revealed increased expression of *β*-defensin 14, and cytokines IL-10 and IFNγ.

**Discussion:**

The introduction of SFB at hatch as a prophylactic treatment may benefit commercial partners as well as consumers by reducing the incidence of *Enterobacteriaceae* in food animals. Reduction of these bacteria in animals would, in turn, increase animal health, productivity, and safety for consumers. Studies to optimize the treatment for poultry industry applications are ongoing in our lab.

## 1. Introduction

The gastrointestinal tract of animals serves as a reservoir for many bacterial species classified as gut symbionts that provide benefits for the host organism (Chow et al., 2010; Pudlo et al., 2015; Shang et al., 2018). These microorganisms often provide benefits to the host through alterations of the gut pH, production of secondary metabolites, and competitively excluding potentially pathogenic organisms (Redweik et al., 2020a). However, some symbionts have been shown to mechanistically impact the maturation of the gut immune system (Wickramasuriya et al., 2022). Of these microbes, segmented filamentous bacteria (SFB), or specifically *Candidatus Arthromitus,* are a gut-associated symbiont regularly found in numerous vertebrate species, including humans, pigs, mice, rats, turkey, and chickens (Klaasen et al., 1992; Redweik et al., 2020b). SFB are gram-positive, spore forming bacteria, closely related to *Clostridia* species that intimately bind to the host epithelium in the ileum of the gastrointestinal tract, as demonstrated in mice (Umesaki et al., 1995). SFB have drawn the attention of researchers owing to their unique morphology, lifecycle, and intimate relation with the host (Hedblom et al., 2018).

In contrast to enteric pathogens, which cause pathology by triggering an inflammatory response following binding to the gut epithelium, SFB drive the maturation of the gut immune system by influencing the production of antimicrobial peptides (AMPs) and the differentiation of naïve CD4^+^ T cells to form CD4^+^ T_H_17 cells (Ivanov and Littman, 2010). Induction of host immune maturation impacts the overall ability of both the innate and adaptive immune systems (Klaasen et al., 1992; Wickramasuriya et al., 2022). Triggering of adaptive immune maturation can provide extraintestinal protection and impact the immune system systemically (McAleer et al., 2016).

A significant amount of research has been accomplished utilizing defined mouse models and mice mono-associated with SFB (Klaasen et al., 1991; Umesaki et al., 1995; Atarashi et al., 2015; Schnupf et al., 2015, 2017; Shi et al., 2019). However, direct studies with SFB are hampered greatly due to the inconsistent ability to culture SFB readily *in vitro* in pure culture (Schnupf et al., 2015). In addition, SFB are host specific, and SFB isolated from mono-associated mice will not colonize any other hosts (Chen et al., 2017). This fact has greatly hindered translational studies to understand how SFB impact the immune systems of different host animals. Specifically, research on the relationship between SFB and the immune system of the chicken gut is severely lacking.

Recently, our lab has demonstrated the ability to prepare a chicken SFB-enriched inoculum that enables the SFB colonization of the ileum of layer chickens that would normally not receive SFB from their environment, due to their hatching in a clean environment and not in contact with hens (Redweik et al., 2020b). As tested in chicken ileum explants, this colonization resulted in measurable immunometabolic changes in the innate (Toll-like receptor, JAK–STAT), adaptive (T/B cell receptor, T_H_17 differentiation), and pathways like the mammalian target of rapamycin (mTOR) pathway in the gut immune system and increased ability to kill *Salmonella in vitro* (Redweik et al., 2020b). Therefore, *in vivo* testing is warranted, and the objectives of this study are to (1) prepare an optimized form of the SFB inoculum, (2) test the ability to increase gut immune maturation, and (3) antimicrobial activity against resident *Enterobacteriaceae* and *Salmonella* challenge in chickens *in vivo*.

## 2. Materials and methods

### 2.1. Ethics statement

Animal experiments were approved by Iowa State University Institutional Animal Care and Use Committee, Log #s 18–386 and 19–072. One-day-old male and female specific pathogen-free (SPF) White Leghorns (VALO; Adel, IA) were used in this study. Animal enrichments were added to open floor pens to minimize stress during experimental procedures. Animals were fed Purina Organic Starter Grower Feed with no antibiotic or probiotic supplementation. Euthanasia techniques (CO_2_ asphyxiation) follow the American Veterinary Medical Association Guidelines (2013) (AVMA guidelines for the euthanasia of animals, 2020).

### 2.2. SFB inoculum preparation

Methods were described previously for the enrichment of ileal spores with minor modifications (Umesaki et al., 1995; Redweik et al., 2020b). Briefly, scrapings from the distal ileum of two-week-old commercial pullets (*n* = 10) were pooled in Phosphate-Buffered Saline (PBS) (3 mM EDTA). Pooled scrapings (*n* = 10) were then treated with chloroform (3% of total solution V/V). The inoculum was allowed to settle at room temperature (RT) for 10 min before the aqueous layer was transferred to a fresh tube. Residual chloroform was evaporated under CO_2_ (Wang et al., 2022). The entire solution was then pelleted at 4500 x g for 15 min. Solutions were not filtered to preserve SFB filaments in the preparations (Schnupf et al., 2015). The pellet was resuspended in a peptone-glycerol (1% peptone W/V, 15% glycerol V/V) solution and stored at −80°C until use in animal experiments. The suspensions were screened on blood agar to ensure the absence of culturable microbes.

### 2.3. qPCR quantification of ilea-associated SFB

Total DNA was extracted from intestinal scrapings utilizing a phenol-chloroform DNA extraction protocol (Gomes-Neto et al., 2017). SFB were enumerated by qPCR quantification of the 16S rRNA gene sequence using the primer pairs: SFB-specific F: 5’-AGGAGGAGTCTGCGGCACATTAGC-3′; and the universal R: 5’-TCCCCACTGCTGCCTCCCGTAG-3′ (Schnupf et al., 2015). The Maxima SYBR/Green master mix (Thermo Fischer, Waltham Massachusetts, United States, Catalog # K0223) was used with reaction conditions consisting of 0.1 mM primers, and 10 ng of template DNA. Samples for qPCR were run in duplicate utilizing a QuantStudio 3.0 thermocycler (Thermo Fischer, Waltham Massachusetts, United States). An SFB standard curve was created by ligating the SFB specific PCR amplicon into the pCR2.1 vector following manufacture instructions (Thermo Fischer, Waltham Massachusetts, United States, Catalog # K202040). The ligated pCR2.1:16S^SFB^ vector was expressed in TOP10 *E. coli,* and plasmid DNA was extracted using the high-speed plasmid mini kit (IBI Scientific, Dubuque, Iowa, United States). The concentration of extracted plasmid DNA was determined using a Qubit 3.0 fluorometer (Thermo Fischer, Waltham Massachusetts, United States). The SFB standard curve was prepared by serially diluting the quantified plasmid stock in tenfold dilutions, and linear regression was performed using GraphPad Prism Software (Version 9.5.1; GraphPad, San Diego, CA). Calculations to convert the cycle threshold to colony forming unit per gram (CFU/g) were performed as described (Gomes-Neto et al., 2017).

### 2.4. Gram-stain and fluorescent *in situ* hybridization identification of SFB

Fluorescent *in situ* hybridization (FISH) was performed based on methods described previously (Haroon et al., 2013; Batani et al., 2019) with minor modifications. Briefly, stored ilea scrapings were pelleted at 13,000 x g for 5 min and washed 2 times with PBS. After the second wash, pellets were resuspended in 50 mL transformation buffer (100 mM CaCl_2_, 30 mM MnCl_2_, 20 mM MgCl_2_, 100 mM potassium acetate, and 10% glycerol V/V). Ilea scrapings were then supplemented with 2,000 ng/mL of the SFB-specific probe (GGG TAC TTA TTG CGT TTG CGA CGG CAC) corresponding to position 801–827 in the universal 16S rRNA gene sequence of SFB (Accession number: X87244) (Liao et al., 2012). The SFB-specific probe was 6-Carboxyfluorescein (6-FAM) labeled (Thermo Fischer, Waltham Massachusetts, United States). After hybridization and washing, FISH-tagged SFB were resuspended in PBS and stored at 4°C for microscopy (Batani et al., 2019). Fluorescence microscopy was performed utilizing a Leica DM4 B fluorescent microscope with L5 filter cube (excitation 480/40 nm) for the detection of the SFB probe (Leica, Wetzlar, Germany).

### 2.5. Pilot inoculation experiment

Day-old birds were distributed into 2 pens (*n* = 18 birds/pen) in the same room. Immediately after placement, birds were orally inoculated with sodium bicarbonate and either 50 μL chloroform-treated scrapings (SFB) or 50 μL PBS (CON). The SFB inoculum contained 10^4^ SFB as determined by qPCR. Food and water were provided 30 min after treatment. Feces from three birds randomly in each pen were collected at days 11 and 14 post SFB treatment to monitor levels of total *Enterobacteriaceae*. Birds (*n* = 6) were euthanized on 2-, 5-, and 16-days post inoculation *via* CO_2_ asphyxiation. To assess levels of SFB, scrapings of the distal ileum were performed, as before, without chloroform treatment and stored in peptone-glycerol at −80°C for future experiments.

### 2.6. *Salmonella* Typhimurium UK-1 χ3761 challenge

Results from the pilot study showed SFB cross-contamination of the CON group through the environment. To address this issue, in this second experiment, birds were distributed into 2 pens (*n* = 12 birds/pen) in separate rooms. Immediately after placement, birds were orally inoculated with sodium bicarbonate and either 50 μL of chloroform-treated scrapings (10^4^ CFU SFB) or 50 μL PBS (CON). At day 4, the birds were orally challenged with 200 μL of nalidixic acid resistant χ3761 (10^7^ CFUs) in PBS. Feces were collected aseptically on 7-, 10-, and 14-days post inoculation (dpi), resuspended in PBS, and plated on MacConkey agar with and without nalidixic acid to track χ3761 and total *Enterobacteriaceae,* respectively. At 16 dpi all birds were euthanized. Intestinal tissues, including contents (ileum and cecum), and extraintestinal tissue (spleen) were aseptically collected, homogenized in PBS, and plated on MacConkey agar with and without nalidixic acid for bacterial enumeration. The limit of detection (LOD) was calculated for each sample as described (Ott et al., 2020). Samples that did not show growth were enriched overnight in 500 μL Luria Bertani (LB) broth with 0.1% glucose at 37°C and re-plated. If no growth was detected after enrichment, these values are displayed as 0 CFU/g tissue. Levels of SFB in the distal ileum were assessed *via* qPCR as in the pilot study.

### 2.7. RT-qPCR assessment of host-derived antimicrobial peptide and cytokine gene expression in the distal ileum

Total RNA was extracted from flash-frozen ilea tissues using RNAzol® RT according to manufacture instructions (Molecular Research Center, Cincinnati, OH, United States, Catalog # RN190) and RNA concentration and quality were assessed *via* a Nanodrop 2000 (Thermo Fischer, Waltham, Massachusetts, USA). Reverse transcription assays were performed *via* the High-Capacity cDNA Reverse Transcription Kit according to manufactures instructions (Thermo Fischer, Waltham Massachusetts, United States, Catalog # 4374966). The RT-qPCR assays were performed using a QuantStudio 3.0 thermocycler (Thermo Fischer, Waltham Massachusetts, USA) and cycling conditions as described (Redweik et al., 2021). Genes encoding host-derived AMPs (b-defensins 12 and 14, cathelicidin b1 and Fowlicidins 1–3) and cytokines (IL-2, 6, 10, 17, and IFNg) were assessed using primers described in Table 1. Differences in gene expression were determined *via* 2^-▵▵^method using the gene encoding glyceraldehyde 3-phosphate dehydrogenase (GAPDH) as a stably expressed housekeeping control gene.

**Table 1:** Primers for reverse transcription qPCR.

Target	Function	Primer sequence	Reference
IL-10	Anti-inflammatory cytokine; ↓ T_H_1 and T_H_2 responses	F:5’-CATGCTGCTGGGCCTGAA-3′ R:5’CGTCTCCTTGATCTGCTTGATG-3’	Shanmugasundaram et al. (2015)
IL-2	Pro-inflammatory cytokine; ↑ T_H_1 responses	F:5’CTGGGAGAAGTGGTTACTCTGA-3′ R:5’CCCGTAAGACTCTTGAGGTTC-3’	Shanmugasundaram et al. (2015)
IL-17	Pro-inflammatory cytokine; ↑ T_H_17 responses	F:5’-ATGGGAAGGTGATACGGC-3′ R: 5’- GATGGGCACGGAGTTGA-3′	Hong et al. (2012)
IL-6	Pro-inflammatory cytokine; ↑ T_H_2 responses	F: 5′- GCTCGCCGGCTTCGA-3′ R:5’-GGTAGGTCTGAAAGGCGAACAG-3′	Weerts et al. (2021)
IFN*γ*	Pro-inflammatory cytokine; ↑Macrophage responses	F:5’GTGAAGAAGGTGAAAGATATCATGGA-3’ R: 5′- GCTTTGCGCTGGATTCTCA-3’	Weerts et al. (2021)
Fowlicidin-1	Antimicrobial peptide	F:5’-GCTGTGGACTCCTACAACCAAC-3′ R: 5’-GGAGTCCACGCAGGTGACATC-3′	Achanta et al. (2012)
*β*-defensin 14	Antimicrobial peptide	F: 5’-ATGGGCATATTCCTCCTG-3′ R: 5’-CTTTGCCAGTCCATTGTAG-3’	Hong et al. (2012)
*β*-defensin 12	Antimicrobial peptide	F: 5’-ACCTTTGTTTCGTGTTCATCTTC-3′ R: 5′- AGGTGCTGCTGCTCTCCA-3’	Hong et al. (2012)
Cathelicidin b1	Antimicrobial peptide	F: 5’-CCGTGTCCATAGAGCAGCAG-3′ R: 5’-AGTGCTGGTGACGTTCAGATG-3′	Achanta et al. (2012)
Fowlicidin-2	Antimicrobial peptide	F: 5’-CAAGGAGAATGGGGTCATCAG-3′ R: 5′- CGTGGCCCCATTTATTCATTCA-3′	Achanta et al. (2012)
GAPDH	Housekeeping Gene	F: 5’-GCACGCCATCACTATCTTCC-3′ R: 5’-CATCCACCGTCTTCTGTGTG-3′	Achanta et al. (2012)

### 2.8. Statistics

Statistical analyses of the data were performed using the GraphPad Prism software (Version 9.5.1; GraphPad, San Diego, CA). Differences in total *Enterobacteriaceae* in feces or SFB quantification in ilea scrapings were measured at each timepoint using Student’s *t*-test. Analyses of differences between either *Salmonella* or total *Enterobacteriaceae* were determined in each organ tested using Student’s *t*-test with Holm-Šidák’s correction. Mann–Whitney *t*-tests were utilized to analyze all RT-qPCR data with Holm-Šidák’s correction.

## 3. Results

### 3.1. SFB-based treatment resulted in colonization of the distal ileum in both animal experiments

**Figure 1:**
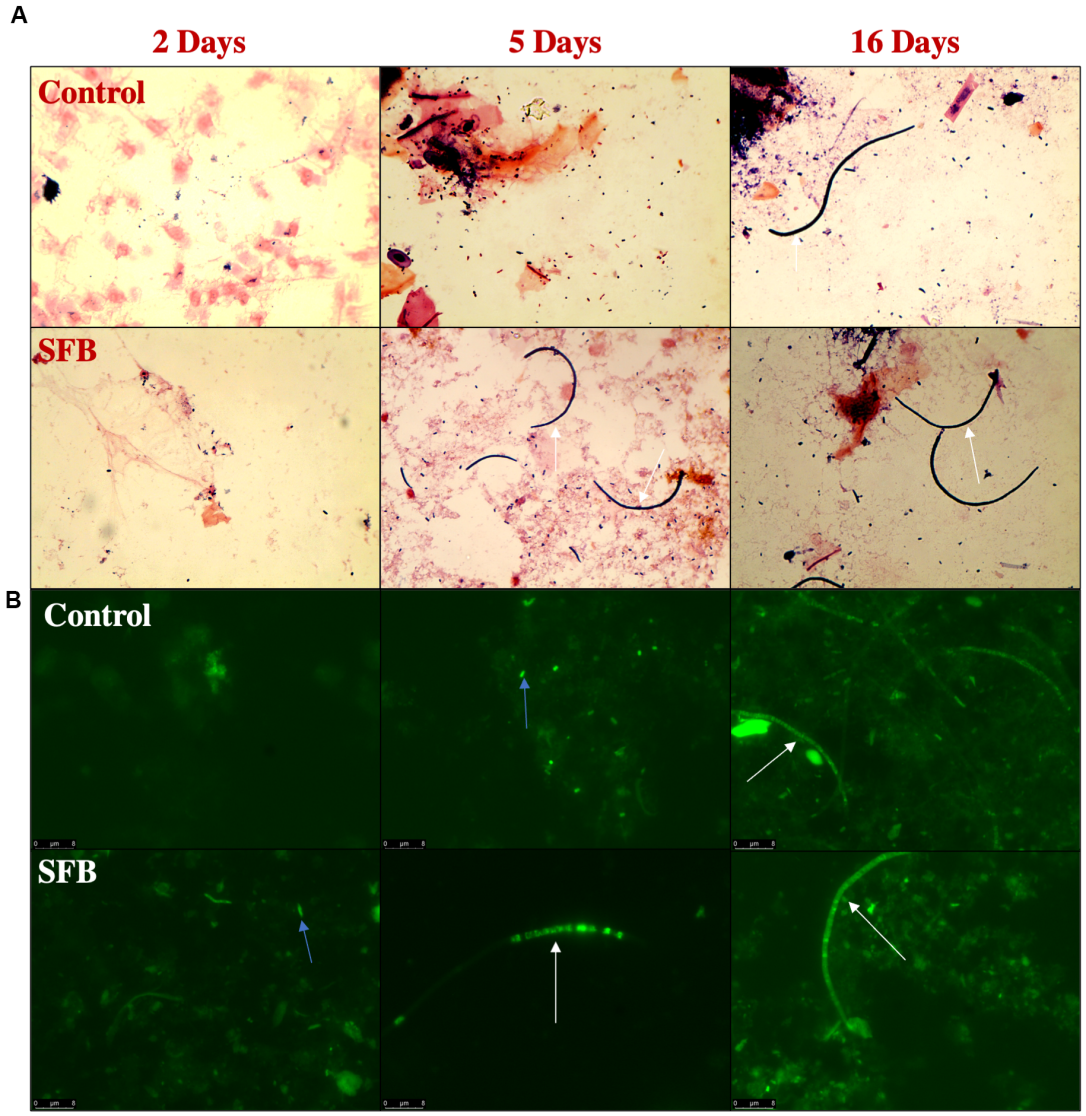
Detection of Segmented Filamentous Bacteria (SFB) *via* Gram-Staining and FISH Microscopy in the Pilot Inoculation Experiment. Representative images of ilea scrapings analyzed by gram-stain **(A)** and FISH microscopy **(B)** demonstrate the inability to detect SFB filaments in Control birds prior to 16-days post inoculation (dpi) and presence of filaments in SFB treated birds after 5 dpi. Arrows indicate intracellular offspring (blue) or filamentous SFB (white).

**Figure 2:**
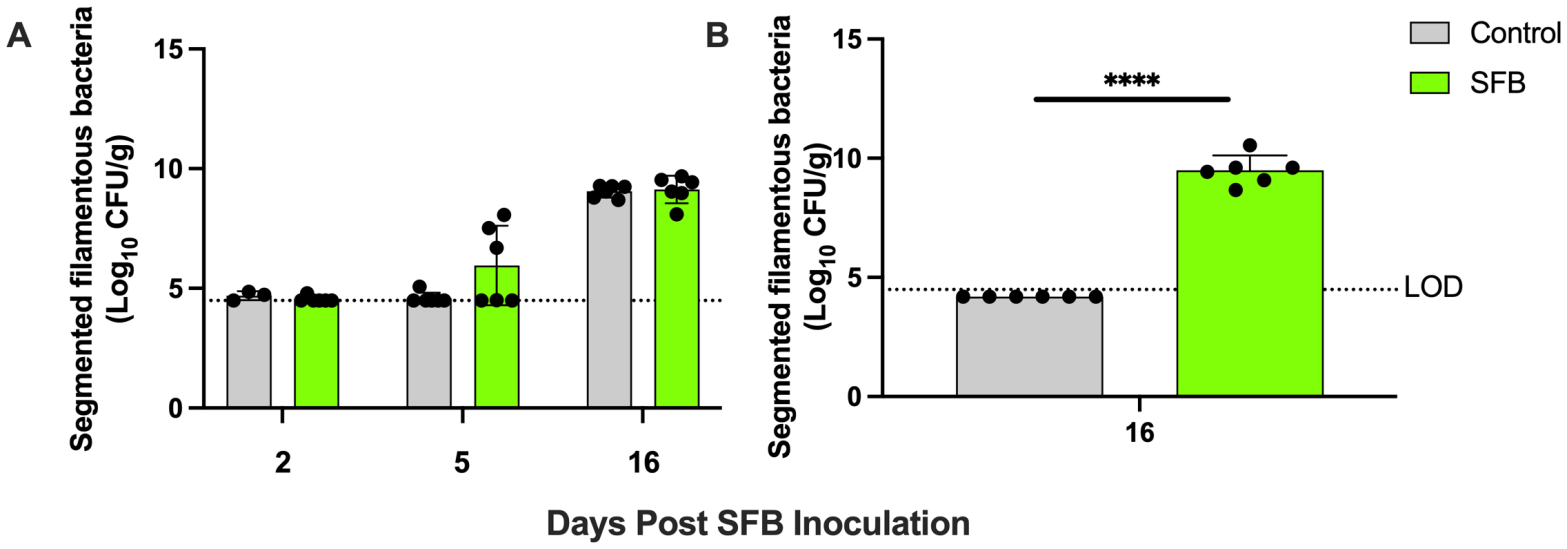
Ilea-associated Segmented Filamentous Bacteria (SFB) Quantification. Log_10_ CFU/g of SFB detected in ilea scrapings calculated by qPCR in **(A)** the pilot inoculation experiment at respective 2-, 5-, and 16- days post inoculation (dpi) and **(B)** *Salmonella* challenge study at final necropsy (16 dpi). ^****^, *p* < 0.0001. Dots indicate individual birds. The horizontal dashed line represents the limit of detection (LOD). Samples that did not demonstrate amplification in qPCR assays were set to half of the LOD.

**Figure 3:**
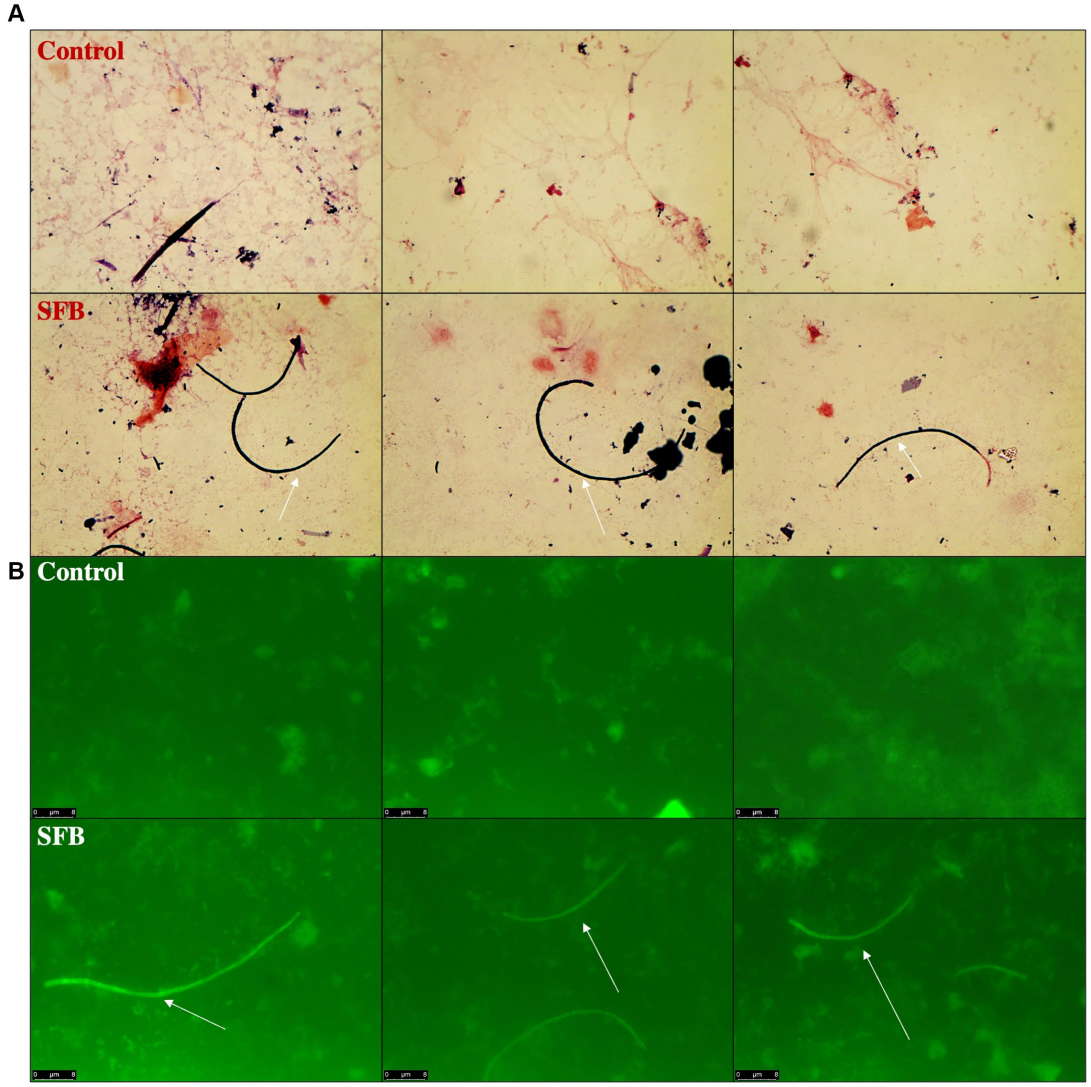
Detection of Segmented Filamentous Bacteria (SFB) *via* Gram-Staining and FISH Microscopy in *Salmonella* Challenge Study. Representative images of ilea scrapings analyzed by gram-stain **(A)** and FISH microscopy **(B)** demonstrate the inability to detect SFB in Control birds and presence of SFB in SFB treated birds at final necropsy (16-days post inoculation). White arrows indicate filamentous SFB.

Through gram-staining and FISH microscopy, we detected SFB filaments in only SFB birds at 5 days post inoculation (dpi). Small holdfast-like morphologies were detected in both SFB and CON groups 2 dpi. Large filaments were found in all birds at 16 dpi (Figure 1). qPCR investigation of the levels of ilea-associated SFB, demonstrated low levels of SFB in all birds at 2 dpi. At 5 dpi, 50% (3/6) of SFB birds demonstrated a large expansion of SFB. At 16 dpi, all birds demonstrated colonization with SFB with no differences between CON and SFB birds (Figure 2A). In the *Salmonella* challenge study, housing CON and SFB birds in different rooms resulted in the colonization of SFB in treated birds only. Gram-strain and FISH microscopy of scrapings from the distal ileum in SFB birds at 16 dpi demonstrated an abundance of filamentous SFB (Figure 3). CON birds showed a complete lack of SFB. Furthermore, SFB-specific qPCR demonstrated a significantly higher (*p* < 0.0001) amount of SFB in SFB birds compared to CON (Figure 2B). Specifically, all CON birds demonstrated a lack of SFB amplification in qPCR assays.

### 3.2. SFB-based treatment reduced total *Enterobacteriaceae* in feces and *Salmonella* χ3761 in the ceca

**Figure 4:**
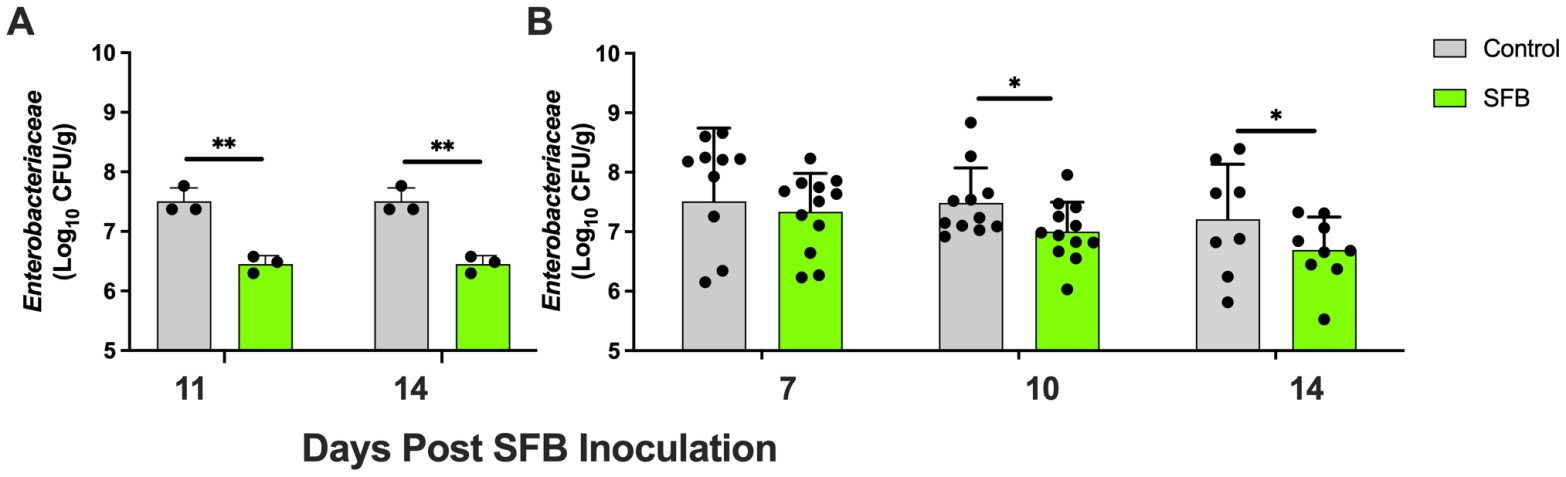
Fecal Tracking of Total *Enterobacteriaceae*. Total *Enterobacteriaceae* in feces in the pilot inoculation experiment at 11- and 14-days post inoculation (dpi) **(A)** and in the *Salmonella* challenge at 7-, 10-, and 14-dpi **(B)**. ^*^, *p* < 0.05; ^**^, *p <* 0.01. Each dot represents a separate bird tested.

**Figure 5:**
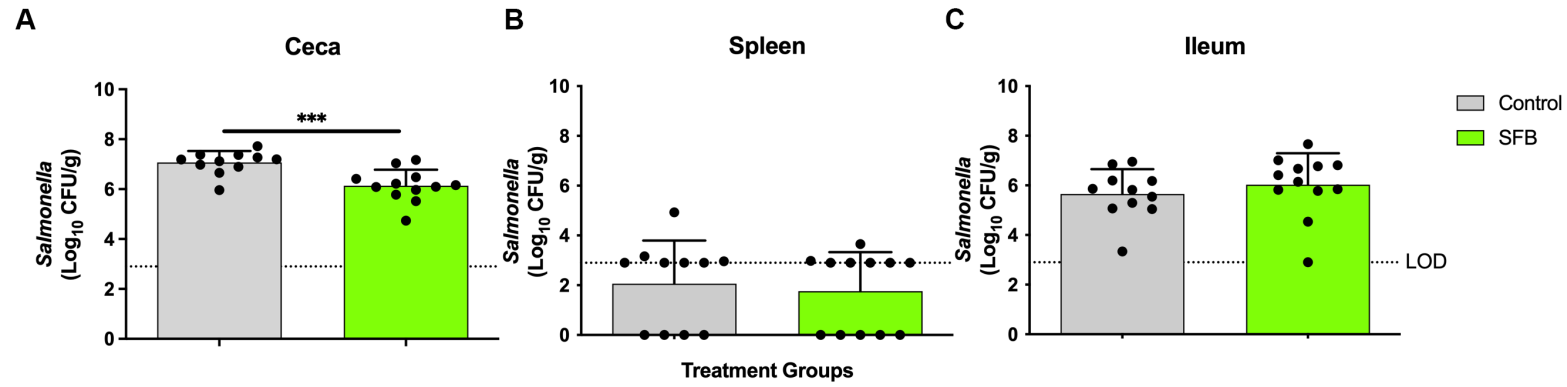
*Salmonella* Enumeration at Final Necropsy. Total *Salmonella* enumerated in the **(A)** ceca **(B)** spleen and **(C)** ileum at final necropsy, 16-days post inoculation. ^***^, *p* < 0.001. Dots indicate individual birds. The horizontal dashed line represents the limit of detection (LOD). Samples that remained negative after enrichment were assigned a 0 value.

**Figure 6:**
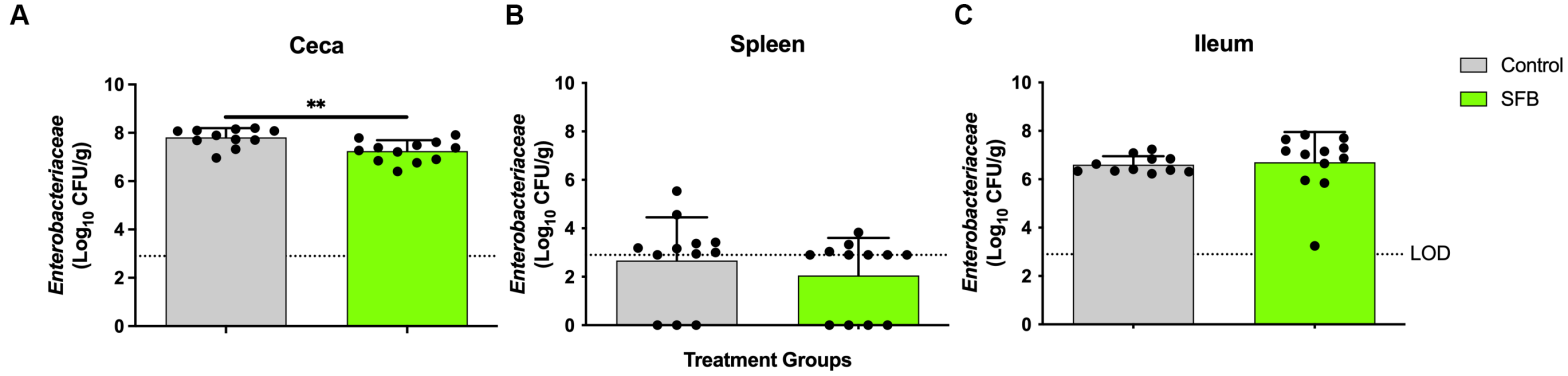
Total *Enterobacteriaceae* Enumeration at Final Necropsy. Total *Enterobacteriaceae* enumerated in the ceca **(A)**, spleen **(B)**, and ileum **(C)** at 16-days post inoculation (dpi). ^**^, *p* < 0.01. Dots indicate individual birds. The horizontal dashed line represents the limit of detection (LOD). Samples that remained negative after enrichment were assigned a 0 value.

In the pilot study, at days 11 and 14 post-inoculation, SFB birds demonstrated significantly lower levels of *Enterobacteriaceae* in feces compared to CON birds tested (*p* < 0.01; Figure 4A). This was further confirmed in the *Salmonella* challenge, where the introduction of SFB significantly reduced the amount of *Enterobacteriaceae* detected in the feces (*p* < 0.05) at 10 and 14 dpi (Figure 4B). At the final necropsy (16 dpi) *Salmonella* χ3761 was detected in all organs tested (Figure 5). The CFU/g tissue of *Salmonella* χ3761 was significantly reduced (*p* < 0.001) in the ceca of SFB birds compared to CON (Figure 5A). Furthermore, the CFU/g tissue of total *Enterobacteriaceae* were reduced in the ceca (*p* < 0.01) of SFB birds compared to CON (Figure 6A).

### 3.3. SFB-based treatment induced differential gene expression of host-derived AMPs and cytokines in the ilea

**Figure 7:**
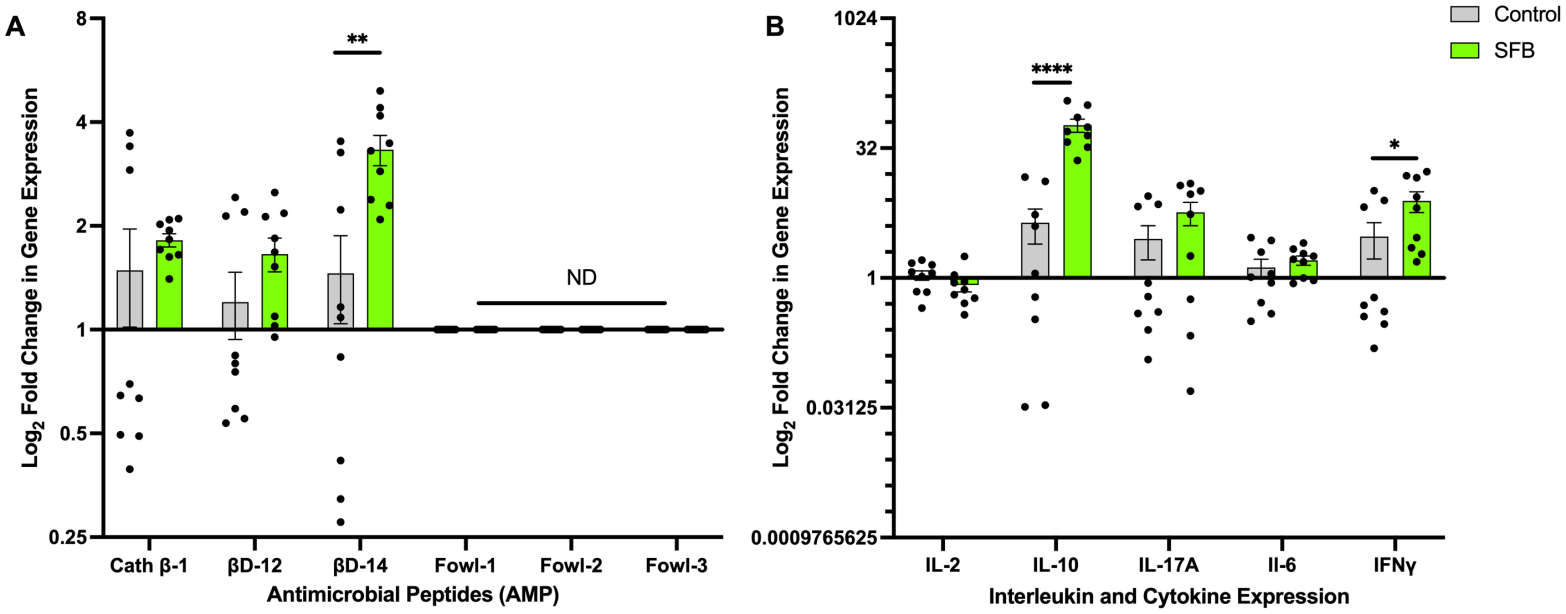
Changes in Antimicrobial Peptide and Interleukin Gene Expression. Log_2_ fold change in gene expression of select antimicrobial peptides (AMPs) **(A)** and interleukins **(B)**. ^*^, *p* < 0.05; ^**^, *p* < 0. 01; ^****^, *p* < 0.0001. ND denotes no amplification detected.

To determine if treatment with SFB preparations results in transcriptional changes in genes of key host-derived AMPs and cytokines, the Log_2_ fold changes in gene expression were measured *via* RT-qPCR. Increased gene expression of the AMP *β*-defensin 14 (*p* < 0.01; Figure 7A), regulatory cytokine IL-10 (*p* < 0.0001; Figure 7B), and cytokine IFNγ (*p* < 0.05; Figure 7B) was observed in the SFB group compared to CON (Figure 7A). There were no significant differences detected in any other AMP or cytokine tested (Figures 7A,B).

## 4. Discussion

Although the lifecycle of SFB and its interaction with the host have been studied in mice, limited studies have been reported on the role of these bacteria in agriculturally relevant animals, such as chickens. A substantial amount of research in mice has demonstrated the key role that SFB play in the maturation of the mouse gut immune system in both the innate and adaptive immune responses (Schnupf et al., 2013; Atarashi et al., 2015; Farkas et al., 2015). Because of the host-specificity of SFB (Chen et al., 2017), our lab has previously studied the interaction of chicken SFB with their host and demonstrated the ability of our chloroform-treated inoculum to promote colonization of the distal ileum with SFB (Redweik et al., 2020b). Identification of the specific role that SFB play in the limitation of pathogen colonization of the gut of chickens will facilitate possible probiotic treatment options in the place of antibiotic intervention.

It is suggested that the lifecycle of SFB is propagated through the adhesion of spores or holdfast cells to the host epithelium in the ileum (Chase and Erlandsen, 1976; Pamp et al., 2012). The transmission of SFB in mammalian hosts is done, in part, thanks to vertical transmission from the mother. However, in the chicken production environment, the eggs are separated from the layer hens and disinfected, thus negating the opportunity for this vertical transmission (Smith, 2019). Our inoculation strategy demonstrates the ability to introduce chicks at day-of-hatch with SFB that propagate to full length filaments in the distal ileum. Furthermore, our data show that birds that do not receive SFB directly *via* inoculation can receive them through shed SFB that are spread throughout the environment. However, in the pilot study, filamentous SFB were only detected in SFB-treated birds at 5 dpi. Birds in the Control group that did not initially receive inoculation of SFB likely began to acquire SFB through environmental spreading at this time point. Based on the lifecycle of SFB, the filamentous form at 5 dpi suggests that the SFB had bound to the host epithelium and progressed through their lifecycle (Pamp et al., 2012). Early SFB colonization is important for the maturation of the gut immune system as although both groups were eventually colonized with SFB in the pilot study, the reduction of *Enterobacteriaceae* was only detected in the SFB-treated birds. When birds were segregated into separate rooms, birds that did not receive SFB on day of hatch remained SFB-negative throughout the entire experiment. These results demonstrate the importance of oral treatment of birds with SFB.

The presence of SFB causes shifts in the gut microbiota in humans, including a reduction of some *Enterobacteriaceae* (Chen et al., 2018). In addition, SFB enhance the production of AMPs from intestinal epithelial cells *in vitro* and in SFB-inoculated germ-free mice (Schnupf et al., 2015). AMPs prevent bacterial pathogens from inducing attachment derived inflammation and lesions (Gubatan et al., 2021; Nazeer et al., 2021). Production of AMPs also prevents SFB from overgrowing and causing pathology in the host (Flannigan and Denning, 2018). Our RT-qPCR data demonstrate that the colonization of SFB in the distal ileum of layer hens triggers the production of AMPs in the ileum. Specifically, we establish that colonization of SFB triggered a log_2_ fold increase in gene expression of *β*-defensin 14. Recent *in vitro* testing on chicken-derived cathelicidins show significant bactericidal effects against various *Enterobacteriaceae* (Veldhuizen et al., 2013). Avian *β*-defensins like *β*-defensin 12 and 14 possess broad-spectrum antimicrobial, LPS neutralizing, and chemotactic properties (Shao et al., 2016). We observed an upregulation of AMP expression in ilea tissue, which may partly elucidate the mechanism by which the reduction of *Enterobacteriaceae* was observed in SFB treated birds.

The up-regulation of IL-10 in the distal ileum of SFB colonized birds suggests that SFB are promoting an anti-inflammatory environment in the gut while synergistically eliminating *Salmonella*. Normally, the expression of IL-10 is linked to continued systemic infection in chickens (Haghighi et al., 2008; Shanmugasundaram et al., 2015; Lalsiamthara and Lee, 2017). However, we did not detect any differences in *Salmonella* (Figures 5B,C) or *Enterobacteriaceae* (Figures 6B,C) load in the ileum and the spleen of CON or SFB birds. In addition, our recent study in chickens, demonstrated that SFB-associated inflammation was non-pathological (Redweik et al., 2020a,b), similar to what has been previously shown in mice (Davis and Savage, 1974; Flannigan and Denning, 2018; Shi et al., 2019). Although we did not observe a significant increase in the expression of IL-17A in SFB-treated birds, only one time point was used for gene expression evaluation. The colonization of SFB has been demonstrated to be directly linked to the differentiation of naïve CD4^+^ T cells to activated T_H_17 cells in germ-free mice (Gaboriau-Routhiau et al., 2009; Lécuyer et al., 2014; Flannigan et al., 2017; Schnupf et al., 2017; Flannigan and Denning, 2018; Shi et al., 2019). T_H_17 cells play a dual role in regulating host inflammation while providing antimicrobial activity against pathogens in the intestine (Blaschitz and Raffatellu, 2010; Crhanova et al., 2011; Huber et al., 2012). Future experiments will aim to track the production of these cytokines throughout the colonization of SFB.

Further, RT-qPCR revealed an increased gene expression of the immunostimulatory cytokine IFNγ in the distal ileum of SFB-treated birds. IFNγ is a cytokine produced by T lymphocytes and natural killer cells throughout the chicken immune system (St Paul et al., 2012; Bagheri et al., 2022). Increased expression of IFNγ in the intestine of *Salmonella enterica* infected chickens has been correlated to a robust T-cell response that results in the rapid clearance of *Salmonella* (Penha Filho et al., 2012; Onuigbo et al., 2018; Ibe et al., 2019). This increase of IFNγ caused by the colonization of SFB is correlated to a significant reduction in *Salmonella* in the ceca. We also demonstrated the reduction of total *Enterobacteriaceae* in the ceca and shed in the feces, which indicates a broad protection against multiple bacteria. As intestinal *Salmonella* was only measured at one timepoint (16 dpi), a continuation of the study may exhibit decreased shedding of *Salmonella* due to this relation with IFNγ expression.

This study demonstrates the ability of an SFB-enriched inoculum to colonize the distal ileum and induce modulations in the innate and adaptive immune responses. Furthermore, we identify the impact the colonization of SFB plays on *Enterobacteriaceae* in the gastrointestinal tract of chickens. Finally, the colonization of SFB indicated protective capabilities against continued *Salmonella* Typhimurium infection in the ceca of layers. By enhancing the ability of layers to clear *Salmonella* infection, SFB may serve as a beneficial organism for egg producers. Current efforts are underway to obtain a pure culture of SFB *in vitro* to optimize dosage *in vivo*. Other studies are underway to assess the protective abilities of SFB against other foodborne pathogens common in the chicken gastrointestinal tract and the overall impact on the complex gut microbiota.

## Data availability statement

The original contributions presented in the study are included in the article, further inquiries can be directed to the corresponding author.

## Ethics statement

The animal study was reviewed and approved by Iowa State University Institutional Animal Care and Use Committee.

## Author contributions

MM conceived and designed the experiments and contributed reagents, materials, and analysis tools. JM-J, LO, and MM performed the experiments and revised the manuscript. JM-J and MM analyzed the data and wrote the manuscript. All authors contributed to the article and approved the submitted version.

## Funding

This research was supported by the United States Department of Agriculture (USDA) National Institute of Food and Agriculture (NIFA) project #026895–0000, USDA- Hatch projects (IOW05700-NC1202 and IOW04202), and Kent corp. to MM. The funders had no role in study design, data collection, and interpretation, or the decision to submit the work for publication. Mention of commercial products is for the sole purpose of providing specific information, not a recommendation or endorsement by the funders.

## Conflict of interest

The authors declare that the research was conducted in the absence of any commercial or financial relationships that could be construed as a potential conflict of interest.

## Publisher’s note

All claims expressed in this article are solely those of the authors and do not necessarily represent those of their affiliated organizations, or those of the publisher, the editors and the reviewers. Any product that may be evaluated in this article, or claim that may be made by its manufacturer, is not guaranteed or endorsed by the publisher.
